# Endoplasmic reticulum stress and mitochondrial dysfunctions in metal-induced neurological pathology

**Published:** 2022

**Authors:** Sophia Cai, Min Woo Kim, Pan Chen

**Affiliations:** 1 Albert Einstein College of Medicine Volunteer Program, Bronx, NY, USA; 2 Rye High School, Rye, NY, USA; 3 GyeongSang National University, Jinju, Korea; 4 Department of Molecular Pharmacology, Albert Einstein College of Medicine, Bronx, NY, USA

**Keywords:** Metal, ER stress, Mitochondrial dysfunction, Neurological disorders

## Abstract

Although essential metal ions are required in the body, neurotoxicity occurs when exposed to a concentration of metal that the body cannot accommodate. In the case of non-essential metals which are important in industry, these elements have the property of causing neurotoxicity even at small concentrations. When such neurotoxicity progresses chronically, it can contribute to various neurodegenerative disorders, such as Alzheimer’s disease and Parkinson’s disease. Therefore, research on the relationships between neurotoxicity and metal metabolism are being actively conducted, and some recent research has suggested that the mechanisms of metal-induced neurotoxicity critically involve endoplasmic reticulum (ER) stress and mitochondrial dysfunction. Hence, this mini-review is to summarize some examples of such evidence and raise new questions in attempting to address metal-induced neurotoxicity with ER stress and mitochondria dysfunctions, two important topics for the effects of metals in neurodegenerative diseases. Taken together, to study the molecular programs of integrating ER stress with mitochondrial dysfunction should be an important area of future research for appreciating the mechanisms of as well as developing strategies and targets for metal-induced neurological diseases.

## Introduction

The endoplasmic reticulum (ER) is an important membranous intracellular organelle for functions such as processing immature proteins, regulating post-translational modification including folding, assembly, glycation, and disulfide binding, and controlling intracellular calcium flux [1]. ER stress occurs when ER is incapable of handling these functional demands which are often due to changes in the physiological and pathological environment [1]. As more evidenced in the literature, when ER stress becomes persistent in the nervous system, it plays an important role for the development of various neurological disorders [2]. In fact, many of these diseases can also be related to dysfunctions of mitochondria, which are normally responsible for generating ATP as required for cellular respiration, therefore playing a critical role in energy metabolism, lipid biosynthesis, and calcium homeostasis. In particular, neuron consumes higher energy than other cells, as the brain exhibits a high 20% of body oxygen uptake although it represents only 2% of the body weight, thus, mitochondria dysfunction through processes such as downregulation of electron transport chain and reduction of ATP production can further implicate and complicate neurological disorders. It has been reported that abnormal metal exposure exacerbates mitochondrial dysfunction, ATP depletion, and abnormal ROS production that finally leads to neuronal cell death [3].

The importance of metals in biology cannot be overemphasized, but as human culture enriches and industries advance, environmental pollution increases, and human beings are overexposed to various metals, causing many chronic diseases including cardiovascular, lung, and kidney disease, and so on. Essential metals, known as subset of trace elements, are present in animal and plant cells and play a major role in homeostasis of cellular physiology and environment as well as protein cofactors [4]. Metal homeostasis is important because uncontrolled intracellular concentration of the metal implicates toxicity or various pathologies. Therefore, the concentration of essential metals in the living organism is strictly regulated. Despite the importance of these metals in biological functions, excessive metal can be accumulated throughout the body and becomes injurious to the health of the nervous system.

In general, the mechanisms of how ER stress contributes to neurological diseases are still rather unclear, while the impacts of mitochondrial function have been relatively more detailed. For instance, elevated metal exposure can exacerbate mitochondrial dysfunction, causing ATP depletion and abnormal ROS production that finally lead to neuronal death [3]. As ER stress and mitochondrial dysfunction can co-exist in metal-related chronic diseases, and while both intracellular processes are important for the pathogenesis of neurological diseases [2], here we summarize some recent literature that has attempted to address ER stress and mitochondria dysfunctions in metal-induced neurotoxicity, which could be significant in the pathogenesis of neurological disorders such as Alzheimer’s disease (AD) and Parkinson’s disease (PD). Using three examples of essential metals copper (Cu), iron (Fe) and manganese (Mn), and two examples of non-essential metals lead (Pb) and arsenic (As, a semimetal), we aim to provide a consensus basis of metals in causing neurotoxicity and neurological disorders through ER stress and mitochondrial dysfunction.

## Metal-Induced ER Stress and Mitochondrial Dysfunction in Neurological Disorders

### Examples of essential metals

#### Copper (Cu):

Copper is an essential trace element for maintaining cellular physiology. It is widely distributed in different brain regions, most prominent in the basal ganglia, hippocampus and cerebellum [5]. Cu is required for neurotransmitter synthesis, cellular respiration and regulation of various enzymes including cytochrome c oxidase [5]. Excessive Cu levels can significantly cause and enhance ER stress, resulting in abnormal proteopathy, a hallmark of neurodegenerative diseases due to that ER stress disrupts protein folding, quality control and degradation system. To date, Cu accumulation has been related to neurological diseases such as AD, ALS (amyotrophic lateral sclerosis), HD (Huntington’s disease), PD and WD (Wilson disease) among many others [5]. At molecular level, Cu has been shown to enhance α-synuclein aggregation, a major type of neural molecular changes in PD [6,7]. Additionally, excessive Cu can induce ER stress-mediated intracellular calcium (Ca^2+^) elevation, thereby it may promote neuronal excitotoxicity by disruption of ionic environment [8,9]. Although evidence is still relatively limited, it has been suggested that AD-associated amyloid precursor protein (APP) or amyloid beta (Aβ) aggregation may be accelerated by excessive Cu [10,11]. With regard to mitochondria, Cu is required for redox homeostasis in the mitochondria. On steady state, Cu is involved in the normal functions of Complex IV and Superoxide dismutase 1 (SOD1); but when Cu is overloaded, abnormal elevation of ROS induces the production of free radicals, possibly leading to disruption of cellular respiration or apoptosis. Indeed, increased ROS from mitochondria dysfunction induced by Cu overexposure can contribute to impairments in spatial learning and memory [12]. Cu decreased dopaminergic neuron marker-tyrosine hydroxylase (TH), the rate-limiting enzyme that catalyze dopamine synthesis from L-DOPA, and mitochondrial dysfunction, and decreased superoxide dismutase (SOD), an enzyme involved in an antioxidant defense, and alteration of mitochondrial transmembrane potential [13,14].

#### Iron (Fe):

Iron plays many fundamental physiological functions in the CNS, such as oxygen transportation, mitochondrial respiration, myelin synthesis and neurotransmitter metabolism [15]. However, Fe overexposure can result in Fe accumulation in the brain, which may increase the risk of AD, PD, HD, aceruloplasminaemia and multiple sclerosis [15]. It has been appreciated that Fe accumulation promotes abnormal aggregation of Aβ and α-synuclein [16,17], however, the mechanisms of Fe-induced ER stress have not been well understood, though it is mainly reported in hepatic dysfunction [18,19]. The peptide hormone hepcidin, derived from hepatocytes, is a central regulator of iron homeostasis and is decreased in pathological conditions, resulting in persistent ER stress [20]. Since neurological disorders exhibited decrease of hepcidin expression, recent studies focused on removing excessive Fe through promoting hepcidin expression [21,22], although it remains to be studied whether Fe-induced ER stress is important. In relation with mitochondrial dysfunction, Fe overexposure can lead to increase in ATP depletion, decrease in complex I activity, and apoptosis in cortical neurons [23] as well as ROS increase in SH-SY5Y cells [24]. Fe accumulation in mitochondria is observed in rotenone-induced SH-SY5Y cells, leading to oxidative stress and neuronal cell death [25]. Iron chelator treatment mitigated the accumulation and mitochondrial oxidative damage in the PD models, including Rotenone-treated SH-SY5Y and MPTP-induced mice [25]. Taken together, iron accumulation through direct exposure or secondary production may be a potential cause for ER stress and mitochondrial dysfunctions in leading to neurological disorders.

#### Manganese (Mn):

Mn is an important mineral for development, nutrition, bone growth and immune system as well as brain and nerve function. On the other hand, excess Mn has been frequently related to neurological diseases and disorders [26]. The associations between Mn, ER stress, and neurological disorders have been addressed albert limitedly in recent literature. For example, overexposure of Mn leads to upregulation of ER stress-related genes including FK506-binding protein family and exhibited toxicity in *C. elegans* [27]. Mn facilitates APP aggregation and α-synuclein aggregation, similar to Cu and Fe, but excessive Mn treatment promotes APP expression and processing in 3xTg AD mouse model [28]. Mn promotes ER stress and ER stress-mediated apoptosis through activation of caspase family in the rat striatum and dopaminergic neurons, resulting in parkinsonism phenotype associated with movement disorders [29,30]. Although the relationships of Mn and ER-stress in neurological disorders are clear, further studies are needed to delineate molecular mechanisms of how Mn-induced ER stress in neurological disorders. In addition to ER stress as discussed, Mn-induced toxicity is highly involved in mitochondrial dysfunction and oxidative stress. Mn overload may directly exacerbates ROS, mitochondrial membrane potential, ATP depletion, and mitochondrial fragmentation, leading to apoptosis [31]. The most severe form of Mn exposure, known as Manganism, entails behavioral changes including tremor, difficulty with gait, balance, and coordination, all similar to Parkinsonism. Indeed, Chronic Mn exposure promotes Mn accumulation in mitochondria of dopaminergic neuron in substantia nigra, leading to movement disorders in mice [32]. Since Manganism and Parkinson’s disease may share the etiological mechanisms, chelation-based therapy might be effective to alleviate Mn-induced mitochondrial dysfunction and ROS, major hallmarks of neurological disorders.

### Examples of nonessential metals

#### Lead (Pb):

Pb exists in the environment as a non-essential metal and is well known as a representative heavy metal among environmental pollutants as pollution is getting more severe as industry develops. Pb overexposure leads to CNS dysfunctions including memory impairment, intelligence, and movement disorders [33]. It has been reported that Pb exposure in *Macaca fascicularis* promotes APP and BACE1 mRNA increase, resulting in AD pathogenesis [34]. Recent studies showed that Pb induces ER stress and increases GRP78 and CHOP in HEK293 cells, suggesting that Pb lead to exacerbation of UPR and apoptosis [35]. Pb promotes α-synuclein aggregation and fibrilization, representing a potentially important step in the pathogenesis in PD [36], while ER stress is likely involved but its role needs to be explored. Overall, future study should give insight into specific mechanisms of Pb-induced ER stress in neurological disorders. In terms of mitochondria, they also play an important role in Pb-induced nervous system dysfunction. Through *in vivo* experiments, it is confirmed that Pb exposure can lead to inhibition on the activities of mitochondrial enzymes such as superoxide dismutase and glutathione reductase [37]. When rat offspring were exposed to Pb, mitochondrial morphology in brain neurons was altered [38]. These changes in mitochondrial structure can cause the release of cytochrome c, induce mitochondria-mediated apoptosis, and evoke changes such as cognitive decline and motor deficit [39]. In neuroblastoma SH-SY5Y cells, Pb promotes mitochondria degeneration which leads to ATP depletion and apoptosis [40]. Inhibition of ROS or autophagy activity as a mechanistic target of Pb-induced neurotoxicity [41], but further study of Pb-induced neurotoxicity is needed to correlate mitochondria dysfunction and Pb-induced neurological diseases.

#### Arsenic (As):

As is useful industrially, but additionally used as an alloy such as lead or copper despite its biological toxicity. Arsenic is a toxic metalloid that can easily cross the blood-brain barrier and accumulate in different parts of the brain, including the striatum and hippocampus, which may potentiate the risk of AD and PD [42]. It has been reported that ER stress is a potential regulatory mechanism in As-induced neurotoxicity [43,44]. In fact, Arsenic species deteriorate protein function and activate UPR and ER stress proteins such as IRE1, GRP78, ATF4, and CHOP. The accumulation of neurodegenerative protein including Aβ and α-synuclein might mechanistically involves a role from As-induced ER stress [43,45]. In rat brain, chronic As exposure promotes APP expression and elevates β-secretase (BACE1) activity, leading to memory impairment [46,47]. In As-treated SH-SY5Y neuroblastoma cells, abnormal α-synuclein accumulation and oligomerization have been observed, which leads to proteotoxicity [48]. However, the role of As-induced ER stress in neurodegenerative diseases still remains largely unknown and thus requires future study to uncover the involved regulating mechanisms. Studies have also revealed that As exposure is related to mitochondrial dysfunction and oxidative stress [49]. As is known to alter mitochondrial membrane integrity, reduce membrane potential, disrupt electron transport chain, generate ROS, and inhibit ATP production and DNA repair [42,50]. In As-treated rats, ROS was induced by decreasing the activity of MnSOD in hippocampus and cortex [51]. It can change a fraction of cellular respiration [52] and increases mitochondrial intracellular Ca^2+^ [53], which may lead to neuronal cell death. Counteracting mitochondrial dysfunction and oxidative stress may represent a strategy for therapeutic intervention against arsenic-induced neurological disorders.

## Conclusions

Metals are indispensable in our lives, but when exposed to excessive amounts, they can cause neuropathological and physiological problems, resulting in neurological damages and disorders. Here we described the emerging evidence that implicate the roles of ER stress and mitochondrial dysfunctions in contributing to metal-induced neurotoxicity and subsequent neurological disorders. Since it is difficult to regenerate neurons after neurodegeneration gets initiated, chronic metal exposure will create synergistic effects with aging and other diseases, further accelerating neurological disorders. As lifespan increases, chronic diseases, including metal-induced neurotoxicity will become more frequent. Therefore, further research on the mechanisms of metal-induced neurotoxicity, as well as therapeutic intervention of metal accumulation should be developed. As reviewed in this mini writing, ER stress and mitochondrial dysfunctions are two important intracellular events in linking metal overexposure to neurological consequences. Evolving steps of ER stress and mitochondrial dysfunctions upon metal excess could be dynamically and reciprocally interacting, but the details of these processes remain to be studied. It is much less clear how functional changes in ER stress and mitochondria are integrated in the context of metal excess. In conclusion, to study the molecular interactions of ER stress and mitochondrial dysfunction and therefore targeting the co-mechanism of these processes represent a significant area of future research for understanding and intervening with metal-induced neurological diseases.
